# Predictors of Early Death in Acute Promyelocytic Leukemia

**DOI:** 10.3390/medsci13040300

**Published:** 2025-12-03

**Authors:** Joana Brioso Infante

**Affiliations:** 1 Serviço de Hematologia e Transplantação de Medula, Hospital de Santa Maria, Unidade Local de Saúde de Santa Maria, 1649-028 Lisbon, Portugal; joana.infante@edu.ulisboa.pt; 2 Faculdade de Medicina, Universidade de Lisboa, 1649-028 Lisbon, Portugal; 3 Instituto de Medicina Molecular João Lobo Antunes, 1649-028 Lisbon, Portugal

**Keywords:** acute promyelocytic leukemia, early death, disseminated intravascular coagulation, supportive care, differentiation syndrome, arsenic trioxide, all-trans retinoic acid

## Abstract

Acute promyelocytic leukemia (APL) evolved from the most lethal to the most curable subtype of acute leukemia today, owing to targeted therapy with all-trans retinoic acid (ATRA) and arsenic trioxide. Despite cure rates exceeding 90% and the rarity of relapse or refractoriness, early death (ED)—occurring within 30 days of diagnosis—remains unacceptably high, reaching up to 30% in population-based studies. ED is the major barrier to universal cure, with fatal hemorrhage as the predominant cause, followed by infection, differentiation syndrome, and thrombosis. Patients who survive the initial month generally achieve excellent long-term outcomes. This review synthesizes data from clinical trials and large real-world cohorts to provide a comprehensive overview of the incidence, causes, and predictors of ED in APL. Higher white blood cell count and older age emerge as the most consistently validated predictors, followed by increased IRB/BICcreatinine, low albumin, thrombocytopenia, and coagulopathy, although their predictive value is not uniform across studies. Risk scores such as the Sanz classification, the Österroos ED model, and dynamic disseminated intravascular coagulation (DIC) assessments represent practical tools for identifying patients at high risk of ED. Importantly, ED rates remain significantly higher in real-world populations than in clinical trials, highlighting the impact of age and comorbidities, delayed diagnosis, and barriers to immediate ATRA initiation and supportive care. Addressing ED in APL requires intensified early supportive strategies, physician awareness and education, and rapid treatment initiation. Refinement and validation of predictive models may guide tailored interventions and inform strategies to finally overcome this persistent unmet need.

## 1. Introduction

Acute promyelocytic leukemia (APL) is a subtype of acute myeloid leukemia (AML) with a unique pathophysiology, targeted treatment, and specific disease-related and therapy-related complications [1]. It is a medical emergency that mandates prompt treatment upon disease suspicion, due to the distinctive ability of its malignant cells to induce disseminated intravascular coagulation (DIC) and hyperfibrinolysis [1].

In recent decades, advancements in PML-RARα-targeted therapies such as all-trans retinoic acid (ATRA) and arsenic trioxide (ATO) have significantly altered the prognosis of APL, transitioning it from a disease with very high early mortality and long-term survival rates of less than 50% to becoming the most curable subtype of adult acute leukemia today [2,3]. However, although long-term survival rates now exceed 90% and APL relapse or refractoriness to treatment are very rare, the early death (ED) rate is still significantly high [4], reaching 30% in some real-world studies [5].

The primary drivers of ED are the inherent thrombohemorrhagic diathesis of APL, which predisposes to both hemorrhagic and thrombotic events, as well as specific treatment-related complications such as differentiation syndrome and infections from neutropenia [6]. APL patients who survive beyond the first month of disease will have an excellent long-term prognosis with current therapies [7].

The major unmet need in APL now is how to best support the patient throughout that critical first month, when timely diagnosis, fast access to ATRA and blood products, quick referral to specialized hospitals, differentiation syndrome prophylaxis and treatment, infection control, and refined supportive and monitoring strategies are key to avoid the imminent death risk that APL poses upon diagnosis [8].

The aim of this review is to provide a comprehensive analysis of the incidence and causes of early death in APL, and to provide a detailed overview of clinical and laboratory predictors reported in the literature. Assessing the current knowledge on ED in APL may pave the way for future studies aimed at refining risk stratification and identifying strategies capable of altering the course of the initial phase of the disease.

## 2. Initial Treatment of APL

Patients with suspected APL should be hospitalized immediately due to the high risk of imminent death associated with the underlying coagulopathy [1]. Three simultaneous actions should be undertaken in the first hours when APL is suspected:

(1) Start ATRA therapy immediately, even before genetic confirmation of the diagnosis;
(2) Administer supportive care with specific blood products;
(3) Confirm genetic diagnosis of APL [1].

ATRA is the cornerstone of APL therapy. It is a differentiating agent that binds and degrades the pathological PML-RARα fusion transcript caused by the t(15;17) and consequently unblocks terminal differentiation of the atypical promyelocytes [9].

ATRA’s role (and speed of action) in counteracting the coagulopathy and hemorrhage has been studied in depth. APL’s coagulopathy was shown to be reversed within a median of 4 days for ATRA, while for chemotherapy alone, the median was 7 days [10]. There are a myriad of mechanisms through which ATRA acts towards the resolution of the APL coagulopathy, out of the scope of this review [11]. This quick effect led to the still-standing recommendation, since the 1990s, that ATRA should be administered immediately at first suspicion of disease [12]. This is further supported by retrospective data showing that delays in ATRA administration increase the risk of early death due to haemorrhage [12].

Since treatment with ATRA takes some time to counteract the coagulopathy, additional interventions are required while ATRA and ATO or chemotherapy are acting at the subjacent cellular level of APL. Such potentially life-saving interventions include intensive blood product support (platelet transfusions, cryoprecipitate/fibrinogen, and/or fresh frozen plasma) and avoidance of anticoagulants, antifibrinolytics, and invasive procedures [1].

Currently, the treatment protocol of APL differs according to the Sanz classification risk of the disease, which is based on the total white blood cells (WBCs) in the peripheral blood at diagnosis. A different “pair” for ATRA is recommended depending on risk: arsenic trioxide for non-high risk (WBC < 10,000/µL), and chemotherapy for high risk (WBC > 10,000/µL) [1]. An interim analysis of the APOLLO trial showed ATO plus ATRA is non-inferior to chemotherapy plus ATRA in high-risk APL patients and may become the new standard of care also for high-risk APL once final results from the trial are presented [13].

With the excellent long-term prognosis conferred by the shift to ATO-based treatment, refractoriness to treatment, APL relapse, and therapy-related secondary neoplasms are now increasingly rare. Therefore, the remaining obstacle to cure is ED as the major unsolved issue of APL [7].

## 3. Early Death in APL

ED in APL is usually defined as death from any cause occurring within 30 days of leukemia diagnosis [14], and this is the time point most frequently reported in clinical trials and observational studies [15].

### 3.1. Evolution of ED Rates over Time

Even though a progressive prolongation in overall survival over the decades has been achieved since ATRA and improved supportive care were implemented, this did not translate into a dramatic reduction in ED rates.

The Swedish APL Patient Registry update published in 2017 shows that, despite increased disease awareness by the Hematology community and adherence to early ATRA treatment, ED rates remain very high [16]. The reported ED rate during the years 1997–2008 was 24%, compared to 26% in 2009–2013. Interpreting these rates according to the Sanz risk, the ED rates decreased in the low and intermediate risk patients (19% to 15%) but dramatically increased from 38% to 50% in high-risk patients. The authors argue that current guidelines are insufficient to tackle the threat of ED, since early ATRA and intensive monitoring and care during the first 30 days of diagnosis were unfortunately not enough to decrease ED over time in Sweden [16].

Likewise, a study on a Canadian APL patient population registry showed no trend towards an improving death rate over the 1993–2007 period [17], nor did an analysis of an American patient registry. This American study shows an increase in long-term overall survival over time, reflecting fewer relapses, but ED rates have not decreased in the same proportion. The ED rate was 22.1% in the 1992–1995 period, 14.7% in 1996–2001, and increased again to 17.5% in 2002–2007 [18].

Finally, a single-institution study from Stanford demonstrated a clear improvement in survival over time, with a 61% decline per decade of the risk of death within 3 years of APL diagnosis. This contrasted with a lack of improvement in 30-day mortality from 1977 to 2007, which was reported to average 20% [19]. These are some of the several published population studies pointing to the inability to effectively decrease ED over the last decades despite all the treatment advances in APL.

In an opposing trend, a registry study from Japan evaluated outcomes from patients diagnosed with APL between 1986 and 2015 and demonstrated an improvement in the ED rate from 31% in the 1986–1995 period to 18.7% in the 2006–2015 period [20]. Despite ATRA being used since the 1990s, a reduction in the early death rate was verified only after 2006.

Regarding more recent years, with the advent of first-line ATO in low-risk APL patients, a study from Shanghai reported a gradual improvement in the ED rate from 20.2% (1990–2002) to 10.1% (2003–2012), and later to 7.0% (2013–2020). However, there was no significant decline in the 7-day death rate (6.1 vs. 5.0 vs. 3.6%, respectively), precisely the period in which hemorrhage dominates [21].

The literature consistently shows that ED rates are not declining enough and are not accompanying the positive trend of longer survival that has been achieved in APL.

### 3.2. Incidence of ED in APL

ED rates reported in the literature differ according to whether the studied population arises from a clinical trial or a real-world registry. Table 1 and Table 2 summarize reported ED rates and the main cause of ED in selected clinical trials and real-world studies, respectively, including more than 100 patients.

Comparing Table 1 with Table 2, a staggering difference can be detected between the range of ED rates reported in each. Clinical trials report a significantly lower ED rate compared to population studies at 3–15% vs. 3.7–32%, respectively. This can be explained by a selection bias of trials towards healthier subjects, after excluding patients who die of hemorrhage before enrollment, those so severely ill that urgent treatment out of trial is required, or are unable to give informed consent, or those with comorbidities that meet strict exclusion criteria [40]. Patients who are in such a severe clinical state are still included in real-world population studies, and most will receive adapted treatment outside of a clinical trial protocol.

The European HARMONY platform of registered patients with newly-diagnosed APL between 1999 and 2022 also demonstrated a relevant difference between the ED rates of patients treated in clinical trials (2.2%) versus in real-world clinical practice (8.1%), underscoring the impact of treatment context on early outcomes [31]. The ED rate also differed based on first-line treatment: among patients treated with ATRA plus chemotherapy, the ED rate was 5.2%, whereas in patients treated with ATRA plus ATO, the ED rate was 2.5%, probably reflecting a higher proportion of non-high-risk APL [31].

The real-world studies reported in Table 2 demonstrate a variability in ED rates across different populations, diagnosis decades, and treatment strategies. The much higher ED rates may reflect a significantly older population in real-world studies than the patients selected for clinical trials, with more significant comorbidities and worse overall performance status. Some patients may be referred to the APL-specialized center from distant regional centers, which increases the odds of presentation at the tertiary institution with an already life-threatening hemorrhage [39,41].

As for the diagnosis decade, ED rates of <10% were reported more frequently in studies after the year 2000, reflecting some improvement in early mortality over the decades. In ATO-based treatment cohorts, ED seems to be quite inferior to rates reported in chemotherapy-based treatment studies.

Even harder to estimate is the rate of very early deaths occurring in patients with newly diagnosed APL who expire before having the chance to begin treatment. These cases happen probably due to delays in diagnosis or to a disease presentation with catastrophic hemorrhage; they are underreported in the literature and commonly excluded from clinical trials [40].

Very few studies have reported the number of APL patients who died before receiving ATRA. In the Swedish Acute Leukemia Registry study, among the 29% of patients who died within 30 days of APL diagnosis, 35% never received ATRA, yielding a very-early death (VED) rate of approximately 10.5% [14]. In a multicenter population study from the USA, 5 out of 204 patients died before ATRA administration (2.5%) [12]. A single-institution cohort study from Stanford reported a death before ATRA rate of 2.9% [19], while an Italian single-institution study reported 4.8% [42]. A Chinese population registry reported nine patients (8.9%) who died before receiving APL therapy [37]. A more recent Portuguese single-center study with 104 patients showed a VED rate of 4.8% [41]. These significantly high numbers show that death before APL treatment is frequent and probably underestimated. The characteristics of these patients who are clinically unstable at APL suspicion or deteriorate quickly, who expire before receiving treatment, need to be further studied in order to guide strategies to reduce VED in APL.

### 3.3. Causes of ED in APL

The leading causes of ED in APL are, in the following order, hemorrhage, infection, differentiation syndrome (DS), and thrombosis [6].

The distribution of causes of death follows a temporal pattern: most fatal bleeds occur early in induction treatment (weeks 1 and 2), while ED caused by infection and DS rise in incidence in weeks 3 and 4 [6]. This is because in the first week of APL, the complex coagulopathy is still uncontrolled, giving rise to the clinical manifestations of hemorrhage and/or thrombosis. Infections are more severe during neutropenia aggravated by cytotoxic APL treatment. DS has two peaks of incidence: one in week 1 and the other in week 3 [43].

#### 3.3.1. Hemorrhage

More than two-thirds of patients with APL display hemorrhagic signs and symptoms at disease presentation, related to the hallmark DIC and hyperfibrinolysis state triggered by APL cells [44]. Hemorrhage is the main cause of ED in APL. The most common location of the fatal bleed is intracranial, followed by pulmonary and, very rarely, gastrointestinal or urogenital hemorrhage [6]. Hemorrhage in vital organs in APL patients usually carries a fulminant course, with a study reporting that 69% of patients who died from cerebral or pulmonary hemorrhage expired within 24 h from the onset of the bleed [6].

A median time of 4–7 days from diagnosis to fatal hemorrhage has been reported, but APL-related hemorrhage can still occur until week 4, despite becoming less frequent after week 2 [6,12,14,25].

Blood product transfusions are a cornerstone to prevent and treat hemorrhagic events, with the aim of keeping a platelet count above 30,000–50,000/µL with platelet transfusions, a serum fibrinogen > 100–150 mg/dL with cryoprecipitate or fibrinogen, and an INR < 1.3 with fresh frozen plasma [1].

Antifibrinolytics have been evaluated in clinical trials with the aim of preventing fatal hemorrhage, but trials failed to show benefit, so these drugs are no longer recommended in routine APL care [1].

#### 3.3.2. Infection

Infections are the second most common cause of ED in APL patients, accounting for an estimated 10–28% of EDs [45]. Pneumonia is the most frequent infection in APL patients, followed by septicemia [6]. Pneumonia is also the leading lethal infection [6].

Death due to infection occurs later than hemorrhagic deaths, with a median time of 21 days after APL diagnosis, reflecting the subsequent worsening of APL-related neutropenia due to myelosuppressive cytotoxic treatment [6].

The ATO plus ATRA combination causes significantly less myelosuppression than chemotherapy plus ATRA protocols, and patients treated with this chemotherapy-free combination also have fewer fever episodes and receive fewer days of antimicrobial therapy [3]. As ATO replaced anthracyclines in first-line care of non-high Sanz risk patients, who make up more than half of the APL population, the number of severe infections has very likely strongly decreased.

#### 3.3.3. Differentiation Syndrome

Differentiation syndrome (DS), recognized for the first time in 1992 by Frankel et al. [46], is a unique complication secondary to the differentiating agents ATRA and/or ATO. It is estimated to occur in 2–27% of patients treated with ATRA, and in 7–31% of patients treated with ATO [47].

The etiology of DS is not fully understood. These agents seem to induce a change in the adhesive properties of atypical promyelocytes and to provoke a release of pro-inflammatory cytokines by the differentiating malignant cells [48]. This results in a systemic inflammatory response state together with increased vascular permeability, capillary leak, and endothelial damage, leading to hypotension, tissue hypoperfusion, and ultimately life-threatening multi-organ failure if the DS is left untreated [48].

There are no universally accepted diagnostic criteria for APL DS, which explains the variability in reported incidence rates in the literature. The criteria proposed by the PETHEMA group are dyspnea, unexplained fever, weight gain greater than 5 kg, unexplained hypotension, acute renal failure, a chest radiograph demonstrating pulmonary infiltrates, or evidence of pleural or pericardial effusion. Patients with 4 or more of the following signs and/or symptoms are classified as having severe DS, while 2–3 criteria classify the DS as moderate [49].

There is no evidence derived from clinical trials regarding the treatment of DS. Several retrospective and prospective studies have shown that prompt treatment of DS with steroids dramatically reduced DS-related mortality, which was estimated at 9% in pre-early steroid era studies [48]. Two Spanish clinical trials that incorporated immediate dexamethasone treatment upon DS recognition report very low ED rates due to DS: 1.1% in one trial and 1.4% in the other [6].

Among the most frequent side effects arising from ATRA and/or ATO usage, DS is the most significant. Other complications, such as iatrogenic leukocytosis and hepatic toxicity (ATRA and/or ATO), or pseudotumor cerebri (ATRA-specific), are not major contributors to ED with proper monitoring strategies [50]. QTc prolongation (ATO-specific) could potentially lead to ventricular arrythmias and cardiac arrest, but this risk is mitigated by avoidance of other QT-prolonging agents, regular electrocardiography monitoring, and correction of electrolytes [50].

Regarding DS prophylaxis, the routine prophylactic use of steroids lacks definitive evidence-based support. Different prophylactic steroid drugs and schedules have been used worldwide; while some clinical trials and protocols adopted steroid prophylaxis for all patients [3], others have used prophylaxis only in patients with a WBC count > 5–10 × 10^9^/L at diagnosis [51]. Even though there is no evidence to support the universal use of prophylactic steroids, their use is highly recommended in APL patients with high-risk disease, in those showing a WBC increase after starting ATRA, or with a serum creatinine > 123 mol/L [1,49].

The adoption of standardized diagnostic criteria, prompt dexamethasone treatment, and corticosteroid prophylaxis has all contributed to turning DS into a manageable APL complication, reflected in the currently very low ED rates due to this syndrome [48].

#### 3.3.4. Thrombosis

Thrombosis is a considerably less frequent manifestation of the thrombohemorrhagic diathesis in APL. A cumulative incidence of thrombosis of 8.6% in APL patients was reported [49], and the percentage of EDs due to thrombosis reported in the literature ranges from 4.1 to 9.3% [50].

Arterial thrombosis is more frequent than venous thrombosis during the first 30 days of APL disease [51]. ED from thrombosis is mostly due to arterial cerebral or cardiac ischemic events; ED from pulmonary embolism has also been reported [52].

Thrombosis is not only a consequence of the complex coagulopathy precipitated by the atypical promyelocyte, as described in detail early in this chapter. During treatment, ATRA quickly counteracts the DIC and hyperfibrinolysis of APL, but through its deregulation of procoagulant and fibrinolytic factors, it has been proposed to induce a prothrombotic state [53], which is supported by the fact that more than 60% of thrombotic events reported in the literature occurred during ATRA treatment, although other studies have contradicted this finding [54]. Severe differentiation syndrome [30] and the use of tranexamic acid [55] have also been shown to aggravate the procoagulant state of APL and thus increase thrombosis incidence.

The danger of using anticoagulant, antiaggregant, or fibrinolytic drugs in a patient with thrombosis and overt APL-induced coagulopathy, as well as the heightened risk of hemorrhagic transformation in a patient who develops an acute ischemic stroke, both contribute to the increased risk of mortality of a thrombotic event in a patient with APL [56].

Evidence-based treatment of life-threatening thrombosis occurring in APL patients is lacking in the literature. The current international guidelines on APL management suggest unfractionated heparin (with dose adjustments to platelet number), albeit carefully considering the potential risk of hemorrhagic transformation if the thrombotic event is an acute ischemic stroke [1].

## 4. Predictors of ED in APL

Table 1 and Table 2 summarize the predictors of ED identified in logistic regression analysis across several studies on large APL cohorts from clinical trials and the real-world setting, respectively.

In the selected clinical trials summarized in Table 1, the most commonly identified predictors of ED were higher WBC count (33.3%), older age (27.8%), increased creatinine (11.1%), low albumin (11.1%), lower platelet count (5.6%), coagulopathy (5.6%), and male gender (5.6%).

As for the large real-world studies summarized in Table 2, the most common predictors of ED were similar to the culprits of ED in the selected clinical trials in Table 1. Again, higher WBC count (28.6%) and older age (23.8%) were the most frequently reported predictors of ED, followed by increased creatinine (9.5%), low albumin (9.5%), lower platelet count (4.8%), coagulopathy (4.8%), male gender (4.8%), and fever (4.8%). In addition, an ECOG performance status above 1 (4.8%) and increased peripheral blasts (4.8%) were also reported as predictors of ED.

### 4.1. Age

Increasing age has been identified as a significant predictor in many studies, with older patients exhibiting a higher ED rate [52,53].

For instance, a 1438-patient cohort from the European HARMONY platform, which includes both patients treated in clinical trials and in real-world settings, found increasing age as a continuous variable to be independently and significantly associated with ED [31]. Together with elderly age, a high Sanz risk score was also independently associated with ED in this large study [31].

Older age emerges as a predictor of ED both in studies involving only chemotherapy-treated patients [30,54] and in ATO-treated patients [36]. This may reflect an interplay of poorer prognostic factors, including comorbidities, reduced physiological reserve, and potentially adaptations in standard treatment approaches according to age.

### 4.2. WBC

A higher WBC at diagnosis emerges as a consistent predictive factor of ED across the literature. An elevated WBC count, usually >10,000/µL and indicative of a higher APL tumor burden, has also been associated with increased risk of hemorrhagic events, the leading cause of ED in APL [6,16,42,52,53,55,57,58,59]. This finding aligns with the pathophysiology of APL, where the malignant atypical promyelocytic cells precipitate DIC and uncontrolled hyperfibrinolysis, exacerbating the risk of fatal hemorrhages [60]. The cut-off of >10,000/µL WBC defines high-risk in the Sanz risk classification of APL; while this risk score was designed to identify risk of relapse, in most studies, high-risk APL also associates with ED.

The WBC count at diagnosis may be influenced by the time latency between disease presentation and diagnosis, as well as the APL’s genetic background. Beyond the PML-RARα, APL is characterized by a limited spectrum of recurrent mutations—primarily FLT3, WT1, NRAS, and KRAS—and a near absence of mutations common in other AML subtypes [61]. FLT3 is the most frequently mutated gene, present in 43% of newly diagnosed cases when ITD and point mutations are combined, and is the sole abnormality (besides PML-RARα) in 25% of patients [61]. In 2019, a meta-analysis on the impact of FLT3 mutations in APL outcomes showed that FLT3 mutations (especially ITD) are strongly [61] associated with higher WBC at diagnosis and with an increased risk of induction death (1.5 to 2-fold higher compared with FLT3-wild-type) [62]. A more recent study published in 2021 reported a 90-patient APL cohort treated with ATRA plus ATO, and FLT3-ITD-positive patients had significantly higher WBC counts at diagnosis and were enriched in the Sanz high-risk group [63]. The ED rate findings were consistent with the earlier meta-analysis, with a statistically significant difference in the ED rate between FLT3-ITD+ versus FLT3-ITD wild-type patients (16.7% vs. 1.4%) [63].

However, the research for valid predictors of ED does not identify the same variables consistently, with some studies failing to identify any predictors at all, even in a very large cohort.

As reviewed above, with high WBC count and increasing age as the most consistently validated predictors of ED, the current APL treatment is stratified over the baseline WBC (ATO or chemotherapy as the pair of ATRA) and according to age (elderly patients recommended to be treated with ATO regardless of APL risk) [1]. One would think ED would be less significant if treatment is tailored to these two variables, but it is still unacceptably high worldwide. Uncertainty therefore persists as to what the best independent predictors of ED are before and during induction treatment for APL. Recognizing clinical and laboratory predictors of ED is key to identifying patients at risk to tailor their care accordingly.

### 4.3. Platelets and Coagulation Parameters

The important role of APL’s coagulopathy as the culprit of the largest fraction of ED cases led to studies on the relationship between individual coagulation parameters and ED. In a 248-patient cohort treated with ATRA plus ATO with an ED rate of 16.1%, baseline WBC, prothrombin time (PT), and activated partial thromboplastin time (aPTT) were associated with an increased risk of ED due to thrombo-hemorrhagic events on univariate analysis. On multivariate Cox regression, only PT and aPTT remained as predictors of thrombo-hemorrhagic ED [64]. In addition, in a Surveillance, Epidemiology, and End Results Registry study including 204 patients with APL treated with ATRA, all-cause ED was associated with higher WBC counts, low fibrinogen, prolonged PT, and prolonged aPTT [12].

The role of platelet count as a predictor of ED in APL has yielded inconsistent results across studies. A study from Sweden developed a model for predicting ED, establishing WBC count, age, and platelet count as the most significant predictors [30]. Subsequently, a risk score was built encompassing these three variables, and this score showed superior discriminative power for ED compared to the WBC-based Sanz risk score [30]. While this Swedish study demonstrated a significant association between baseline platelet count and ED, an 813-patient study treated with ATRA plus daunorubicin from Brazil applied this risk model to predict ED in their population, but platelets did not reach statistical significance in their model [35]. This study identified a high white blood cell count and age above 40 years as independent predictors of ED. These discrepancies in the prognostic impact of platelet count may be context-dependent, possibly influenced by differences in study populations, supportive care practices, and treatment protocols.

Lastly, a low baseline serum fibrinogen (<100 mg/dL) was shown to be an independent predictor of ED in both young and older patients with APL treated with ATRA plus ATO in a 409-patient cohort from China [36].

### 4.4. Disseminated Intravascular Coagulation Score

The Disseminated Intravascular Coagulation (DIC) Score of the International Society of Thrombosis and Hemostasis is calculated using the platelet count, PT, fibrinogen, and d-dimers, and a Score of 5 or more is compatible with DIC [65]. Some of these individual standard coagulation parameters included in the score have been shown to predict hemorrhage and ED in APL, as detailed in the previous section.

Early recognition and management of DIC are crucial components of the overall care strategy for APL patients [1]. Therefore, there has been interest lately in studying the applicability of the DIC Score in patients with APL. Three recent studies have explored the potential role of the baseline DIC Score in predicting outcomes in APL patients.

The first study found that the usual cut-off of ≥5 for overt DIC did not associate with ED, but instead a Score ≥6 was significantly associated with ED (12% if score ≥ 6 versus 0% if score < 6) and with both lethal and non-lethal hemorrhage or thrombotic events (35% if score ≥ 6 versus 11% if score < 6) [66]. A study from Turkey identified the DIC Score as a predictor of ED on univariate analysis, but this association lost significant power in the multivariate analysis, which identified only age > 55 and ECOG performance status ≥ 2 as predictors of ED [67]. As the severity of the coagulopathy may change during the early treatment of APL, the evolution of the DIC Score and its association with mortality in APL was assessed in a Portuguese study. In patients succumbing to ED, an improvement of the baseline DIC Score was less frequent than in survivors beyond 30 days, while a worsening DIC Score was significantly more common (30.4% versus 6.4%). On multivariate regression analysis, the independent predictors of ED identified were a worsening DIC Score after 48 h (which conferred almost 8 times higher likelihood of ED) and age > 60 years [68]. The authors suggest intensifying blood product administration in patients whose baseline DIC Score worsens as a preventative strategy to potentially decrease ED [68,69], together with other recommended supportive measures [1].

### 4.5. Mortality Cause-Specific Predictors

Another lens through which predictors of ED can be examined is according to the cause of death. While most studies focus on all-cause ED, a few shed some light on specific causes of mortality. The largest study, from PETHEMA, identified age over 60 years, male gender and fever at presentation as independent prognostic factors for ED due to infection; an Eastern Cooperative Oncology Group (ECOG) performance status above 1 and low albumin as independent predictors of DS-related ED; and abnormal creatinine, increased peripheral blast counts and coagulopathy as independent prognostic factors for hemorrhage-related ED [6].

Severe hemorrhage and hemorrhage-related mortality have been the focus of more studies than for other causes of death in APL. Independent predictors of severe hemorrhage in multivariate analysis vary between these published studies. Proposed predictors have included WBC count, high peripheral blast count, low platelet count, prolonged PT and aPTT, decreased fibrinogen, increased lactate dehydrogenase, increased creatinine, and decreased performance status [6,55,59,70,71,72]. Most of the cohorts from which this data derives are small, with under 200 patients. The largest study to date focusing solely on ED caused by hemorrhage gathered data on 995 patients enrolled in 5 clinical trials that included ATRA in the induction treatment. At 30 days, the incidence of hemorrhagic death was 3.7%, and on multivariate analysis, a high total WBC count (≥20 × 10^9^/L) emerged as the only independent predictor of early hemorrhagic death [53].

### 4.6. Resource-Limited Settings

Recent studies have pointed to the significance of socioeconomic factors and access to healthcare as contributors to ED. Patients from rural or under-resourced settings face barriers to timely diagnosis and treatment initiation [34], which are crucial in preventing ED. The role of early ATRA administration, even before definitive diagnosis, as a strategy to reduce ED underscores the critical window immediately following healthcare admission, where prompt, aggressive management can be lifesaving.

In the clinical trial ECOG-ACRIN EA9131, Jillella et al. investigated whether implementing a standardized treatment algorithm in community hospitals with co-management of patients with experts in APL from academic centers and community oncology practices could reduce 30-day ED [73]. With this collaboration, ED in this study was 3.0%, much lower than historical rates (17–30%) from real-world community hospital cohorts. This trial provides strong evidence that ED in APL can be dramatically reduced by detailed management algorithms, standard of care level of APL-specific therapy, intensive supportive care, physician education, and academic-community partnerships, which will help mirror trial-level outcomes into real-world practice.

In Brazil, delayed diagnosis and limited access to ATRA or blood product support have historically contributed to high rates of ED, which reached 32% [5]. To address these potentially modifiable logistical and socioeconomic factors, the American Society of Hematology established the International Consortium on Acute Promyelocytic Leukemia (IC-APL) in 2004 with the aim of improving outcomes of APL in developing countries, including Brazil, with the adoption of locally adapted diagnosis, treatment, and supportive care recommendations [27]. Implementation of the IC-APL network led to an almost 50% reduction in early mortality and a nearly 30% improvement in overall survival compared with historical controls, achieving OS and DFS outcomes comparable to those reported in developed countries. This endeavour proved that networking, improved supportive care, and application of locally adapted guidelines contributed to improving ED levels approaching what is seen in more resourced settings.

Limited access to ATO in some settings may also contribute to higher ED rates from APL in such regions, where anthracyclines are the only available partner for ATRA. Chemotherapy-based induction is associated with more frequent treatment-related mortality from severe infections, whereas ATO-based induction induces less myelosuppression and fewer ED compared to the anthracyclines plus ATO as reported in the APL-0406 trial [3]. The HARMONY study on a large cohort of APL patients showed lower ED incidence using ATO-based induction versus chemotherapy-based induction, both in clinical trials and in real-world practice [31].

### 4.7. Predictors of Death Before APL Treatment

Finally, only a few studies have focused on predictors of death before treatment in patients with APL. In 2010, an Italian study reported five patients with fatal hemorrhagic complications before starting ATRA plus chemotherapy. Compared with the patients who began APL-specific therapy, these patients more frequently had a delayed diagnosis, altered coagulation values, elevated WBC, a high peripheral blast count, and phenotypic expression of CD2 [42]. In a 104-patient cohort from Portugal, 5 patients expired before starting ATRA [41]. The factors significantly associated with very early death included the identification of a serum creatinine  >  1.5 mg/dL, a DIC Score  ≥  7, a DIC Score  ≥  6 in the second coagulation assessment within 24 h of APL suspicion, and the need for mechanical ventilation at hospital arrival [41]. The difficulty in delivering ATRA safely through a nasogastric tube in mechanically ventilated patients may have contributed to the quick deterioration and demise of these patients, together with the critically ill state in which these patients were when admitted to the hospital.

## 5. Measures to Prevent ED

While the induction treatment of APL patients is dictated by the Sanz risk, with possible adaptations of treatment protocol in elderly patients, standard intensive APL-specific supportive care measures are warranted regardless of APL risk and are recommended by an expert panel from the European LeukemiaNet to reduce ED [1]:

- Immediate administration of ATRA upon APL suspicion and not waiting until the marrow is examined or the PML-RARα is genetically confirmed to begin ATRA.
- Coagulopathy support:
  ○ Administration of fibrinogen or cryoprecipitate if serum fibrinogen < 100–150 mg/dL, fresh frozen plasma or prothrombin complex if PT-INR > 1.3, and platelet transfusion if platelets < 30,000–50,000/µL.
  ○ Monitoring coagulation parameters from one to four times daily until all laboratory and clinical signs of the coagulopathy resolve.
  ○ No routine use of fibrinolytics, heparin, or other anticoagulant therapy since their benefit as routine care in APL is questionable.
  ○ Avoidance of invasive procedures before and during induction therapy because of a high risk of hemorrhage.
- Differentiation syndrome (DS) prophylaxis with corticosteroids.
- Daily evaluation for signs and symptoms of DS to allow for early recognition and immediate treatment with dexamethasone.
- Treatment of leukocytosis to reduce the atypical promyelocyte burden in the peripheral blood, either with idarubicin in high-risk patients, or hydroxyurea, idarubicin, and/or gemtuzumab ozogamicin for low-risk patients who develop leukocytosis under ATRA plus ATO treatment.
- Prophylactic antibiotics and/or antifungals according to local infection control guidelines.

The widespread adoption of these measures, together with APL-targeted treatment, has revolutionized the landscape of this previously most lethal leukemia towards becoming the most curable in adults nowadays. Unfortunately, not all patients will survive the first hazardous month of APL disease despite the correct application of these interventions.

**Figure 1:**
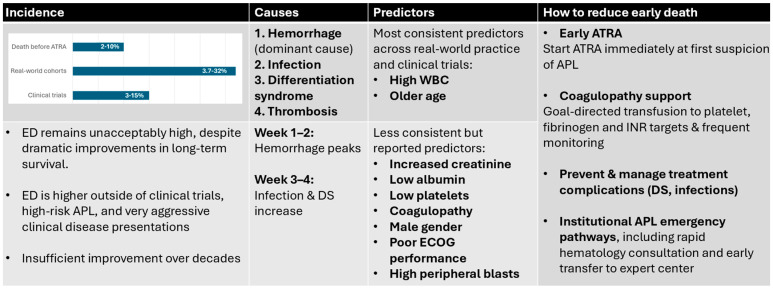
Overview of Early Death in Acute Promyelocytic Leukemia across four domains. Incidence: Reported ED rates vary widely, with the lowest rates observed in clinical trials (approximately 3−15%), substantially higher rates in real-world cohorts (3.7−32%), and a significant proportion of deaths occurring before initiation of ATRA (2−10%). Despite major improvements in long-term survival, ED has shown limited reduction over time and remains disproportionately high in high-risk disease and outside controlled trial settings. Causes: Hemorrhage is the dominant mechanism of ED, particularly within the first 1–2 weeks of presentation, while infection and differentiation syndrome become more prominent contributors in weeks 3–4. Predictors: The most consistent predictors identified across studies are an elevated presenting white blood cell count and older age. Additional reported predictors include renal dysfunction, hypoalbuminemia, thrombocytopenia, coagulopathy, male sex, poor performance status, and high peripheral blast counts. Strategies to reduce ED: Key opportunities to lower ED include immediate initiation of ATRA at first suspicion of APL, intensive and goal-directed transfusion support for treatment of coagulopathy, early recognition and management of treatment-related complications (particularly differentiation syndrome, infection, and QT-related arrhythmias), and implementation of institutional APL emergency pathways which enable rapid access to expert hematologic care and to ATRA/transfusions, as well as critical care support. ATRA—alltransretinoic acid, DS—differentiation syndrome, ECOG—Eastern Cooperative Oncology Group, WBC—white blood cell count.

Figure 1 provides an integrated overview of incidence patterns, mechanisms, predictors, and potential strategies to reduce early death in APL.

## 6. Conclusions

Overcoming ED—either before treatment or during treatment—is the biggest challenge APL clinicians currently have at hand. There are still missing pieces in the puzzle of deciphering the modifiable contributors to ED in APL in order to significantly reduce ED in APL.

The two most systematically identified predictors of ED—higher WBC count and older age—already dictate the ideal APL treatment protocol for each patient with a choice between ATO or chemotherapy plus ATRA, but this stratification did not lead to the eradication of ED in APL. Thus, this highlights a need for validation of new tools that stratify newly diagnosed patients with APL according to their risk of ED. As reviewed, the ED risk score developed by Österroos et al. [65], the Sanz risk, and static or dynamic DIC Score assessments have been shown to be effective and easily available in daily clinical practice to help identify the patients at highest risk of death. The continued refinement of such scores could then inform the creation of tailor-made transfusion protocols and influence the treatment approach and frequency/intensity of monitoring, which would align with the unique risk profile of each patient [65].

The most consistent predictors of ED reported across clinical trials and real-world studies are the same and have a similar distribution. These were, in decreasing order of frequency, higher WBC count, older age, increased creatinine, low albumin, lower platelet count, coagulopathy, and male gender.

In the era of such effective APL first-line therapy, addressing ED requires a multifaceted approach that includes not only the optimization of supportive care and prophylactic strategies but also the improvement of healthcare access to ensure a fast APL diagnosis and rapid access to treatment by healthcare teams specialized in APL care. Wide availability of ATRA in emergency rooms, physician education across Hematology, Emergency Medicine, Pathology, and other areas to recognize suspicious signs of APL, quick referral protocols, and physician-to-physician networking with experts in APL are some recommended measures to globally prevent ED [8].

Future studies should aim to identify additional predictors of ED and to investigate local and global interventions that can mitigate these risks of early hemorrhage, thrombosis, infection, and DS, thereby improving the prognosis for all patients with APL.

## Figures and Tables

**Table 1:** Early death rates at 30 days reported in major clinical trials in APL, including major cause of ED and identified prognostic factors of ED in each study.

Study	Patients	Years	Induction Treatment	ED Rate	Major Cause of ED	Prognostic Factors for ED
Fenaux et al. [22]	413	1993–1996	ATRA + Daunorubicin + ARA-C	7%	Sepsis (42%) CNS hemorrhage (33%)	Older age Higher WBC count
Lo-Coco et al. [23]	1081	1993–2006	AIDA	6%	Hemorrhage (35%) Sepsis (17%)	Higher WBC count Lower platelet count Older age
Burnett et al. [24]	120	1993–1997	ATRA + Dauno/ARA-C	12%	Hemorrhage (36%) Infection (18%)	Higher age Higher WBC count
Lengfelder et al. [25]	142	1994–2005	ATRA + TAD/HAM	8%	Hemorrhage (36%) Infection (18%)	Higher age 99 Higher WBC count
Sanz et al. [26]	426	1996–2002	AIDA	9%	Hemorrhage (64%) Sepsis (28%)	Age > 70 years WBC > 10,000/uL
De la Serna et al. [6]	732	1999–2005	ATRA + Idarubicin	9%	Hemorrhage (56%) Infection (26%)	ED by hemorrhage: abnormal creatinine, increased peripheral blast counts, coagulopathy ED by infection: age > 60 years, male gender, fever ED by DS: ECOG PS > 1, low albumin
Lo-Coco et al. [3]	156	2007–2012	AIDA ATRA + ATO	3%	Differentiation syndrome (50%) Acute ischemic stroke (25%) Infection (25%)	NA
Platzbecker et al. [13]	131	2016–2022	AIDA ATRA + ATO	10% 7%	Intracranial hemorrhage (58%)	NA
Rego et al. [27]	180	NA	ATRA + Dauno	15%	Hemorrhage (48%) Infection (26%)	Higher WBC count Coagulopathy Increased creatinine Low albumin

AIDA—all-transretinoic acid plus idarubicin, ARA-C—cytarabine, ATRA—all-trans retinoic acid, ATO—arsenic trioxide, CNS—central nervous system, CRP—C-reactive protein, DS—differentiation syndrome, ECOG PS—Eastern Cooperative Oncology Group Performance Status, ED—early death, ICU—intensive care unit, LDH—lactate dehydrogenase, NA—not available, WBC—white blood cells.

**Table 2:** Early death rates at 30 days reported in major real-world cohorts in APL, including major cause of ED and identified prognostic factors of ED in each study, ordered by the initial year of the study period.

Study	Patients	Years	Induction Treatment	ED Rate	Major Cause of ED	Prognostic Factors for ED
Li et al. [20]	3212	1986–2015	Not reported	21.3%	Not reported	Older age Earlier diagnosis year Socioeconomic status
Gill et al. [28]	751	1991–2021	ATRA + Dauno Oral ATO + ATRA + Dauno	19%	Differentiation syndrome (45%)	Male gender Age > 50 years WBC > 10 × 10^9^/L Diagnosis in 1991–2009
Park et al. [18]	1400	1992–2007	Not reported	17.3%	Not reported	Age ≥ 55 years Diagnosis in 1992–1995 (vs. 1996–2007)
Altman et al. [12]	204	1992–2009	ATRA + chemotherapy	11%	Hemorrhage (61%)	Higher WBC count Low fibrinogen Prolonged PT Prolonged aPTT
Paulson et al. [17]	399	1993–2007	Not reported	22%	Not reported	Age ≥ 50 years
Rashidi et al. [29]	120	1996–2013	ATRA + chemotherapy	17%	Hemorrhage (55%)	WBC > 10 × 10^9^/L DIC on admission Transfer to ICU
Lehmann et al. [14]	105	1997–2006	ATRA-based	29%	Hemorrhage (41%)	Higher WBC count High LDH High CRP Lower platelet count Increased creatinine
Lehmann et al. [16]	195	1997–2013	ATRA-based	25.1%	Hemorrhage (46%)	Older age Higher WBC count Lower platelet count Increased creatinine
Österroos et al. [30]	301	1997–2020	ATRA + idarubicin + cytarabine	19.6%	Hemorrhage or thrombosis (59.4%)	White blood cell count Lower platelet count Older age
Voso et al. [31]	1438	1999–2022	ATO + ATRA or ATRA + idarubicin	Overall: 5.9% Clinical trial: 2.2% Real-world: 8.1%	Hemorhage Infections	Increasing age High Sanz risk score
Murthy et al. [32]	2962	2000–2014	Not reported	2000–2004: 25.3%; 2005–2009: 20.6%; 2010–2014: 17.1%	Not reported	Older age Earlier treatment period (2000–2009) Male gender Hispanic ethnicity
Zhu et al. [33]	265	2001–2012	ATRA + ATO ± chemotherapy	6.8%	Hemorrhage (66.7%)	Not reported
Jacomo et al. [5]	157	2003–2006	ATRA + chemotherapy	32%	Hemorrhage (66.6%)	Not reported
Dhakal et al. [34]	7190	2004–2015	Not reported	12% (19–40 y: 6%; 41–60 y: 10%; >60 y: 21%)	Not reported	Higher Charlson Comorbidity Index No private insurance Treatment at community center
Silva et al. [35]	813	2005–2020	ATRA + daunorubicin	14%	Hemorrhage (60.5%)	Age ≥ 40 years High white blood cell count
Jin et al. [36]	409	2007–2019	ATO + ATRA	Elderly pts: 23.74%; younger pts: 11.85%	Hemorrhage	Elderly patients: male gender, WBC count > 10 × 10^9^/L, fibrinogen < 1.0 g/L, low albumin levels Younger patients: higher WBC count, lower fibrinogen, and increased creatinine
Zhu et al. [37]	1233	2015–2019	ATRA + ATO in 78%	8.2%	Intracranial hemorrhage (59.4%)	Age ≥ 60 years WBC > 10 × 10^9^/L
Lebon et al. [38]	135	2010–2021	ATRA + CHT (62%) ATRA + ATO (38%)	ATRA + ATO: 10% ATRA + CHT: 22%	Hemorrhage (45.8%), sepsis (41.7%)	Not reported
Infante et al. [39]	135	2017–2024	ATO + ATRA	3.7%	Intracranial hemorrhage (60%) Septic shock (40%)	Older age (age > 75 years)

aPTT—activated partial thromboplastin time, ATRA—all-trans retinoic acid, ATO—arsenic trioxide, CRP—C-reactive protein, DIC—disseminated intravascular diagnosis, ED—early death, ICU—intensive care unit, LDH—lactate dehydrogenase, PT—prothrombin time, WBC—white blood cell.

## Data Availability

No new data were created or analyzed in this study.
